# Transient Receptor Potential (TRP) and Thermoregulation in Animals: Structural Biology and Neurophysiological Aspects

**DOI:** 10.3390/ani12010106

**Published:** 2022-01-02

**Authors:** Karina Lezama-García, Daniel Mota-Rojas, Alfredo M. F. Pereira, Julio Martínez-Burnes, Marcelo Ghezzi, Adriana Domínguez, Jocelyn Gómez, Ana de Mira Geraldo, Pamela Lendez, Ismael Hernández-Ávalos, Isabel Falcón, Adriana Olmos-Hernández, Dehua Wang

**Affiliations:** 1 PhD Program in Biological and Health Sciences, [Doctorado en Ciencias Biológicas y de la Salud], Universidad Autónoma Metropolitana, Mexico City 04960, Mexico; 2192801631@alumnos.xoc.uam.mx; 2 Department of Agricultural and Animal Production, Universidad Autónoma Metropolitana (UAM), Unidad Xochimilco, Mexico City 04960, Mexico; 2212801915@alumnos.xoc.uam.mx (A.D.); 2173025424@alumnos.xoc.uam.mx (J.G.); 2192029639@alumnos.xoc.uam.mx (I.F.); 3 Mediterranean Institute for Agriculture, Environment and Development (MED), Institute for Advanced Studies and Research, Universidade de Évora, Pólo da Mitra, Ap. 94, 7006-554 Évora, Portugal; apereira@uevora.pt (A.M.F.P.); ageraldo@uevora.pt (A.d.M.G.); 4 Animal Health Group, Facultad de Medicina Veterinaria y Zootecnia, Universidad Autónoma de Tamaulipas, Victoria City 87000, Mexico; jmburnes@docentes.uat.edu.mx; 5 Faculty of Veterinary Sciences, Veterinary Research Center (CIVETAN), Universidad Nacional del Centro de la Provincia de Buenos Aires (UNCPBA), CONICET-CICPBA, Arroyo Seco S/N, Tandil 7000, Argentina; ghezzi@vet.unicen.edu.ar (M.G.); palendez@vet.unicen.edu.ar (P.L.); 6 Facultad de Estudios Superiores Cuautitlán, Universidad Nacional Autónoma de México (UNAM), Cuautitlan Izcalli 54714, Mexico; mvziha@comunidad.unam.mx; 7 Division of Biotechnology—Bioterio and Experimental Surgery, Instituto Nacional de Rehabilitación-Luis Guillermo Ibarra Ibarra (INR-LGII), Tlalpan, Mexico City 14389, Mexico; solmos@inr.gob.mx; 8 School of Life Sciences, Shandong University, Qingdao 266237, China; dehuawang@sdu.edu.cn

**Keywords:** TRP, thermoregulation, ion channels, mammals

## Abstract

**Simple Summary:**

In this review, recent discoveries regarding transient receptor potential are discussed and analyzed to comprehend their role in the thermoregulatory mechanisms of animals. Understanding how these receptors are activated and the pathways through which they recognize specific thermal sensations (such as cold, warm, and hot temperatures) will help researchers verify their participation in inflammatory and pathological processes. Research on transient receptor potential and their functions is ongoing, and many current studies are designed to develop therapeutic approaches that will act directly on these receptors to improve the quality of life of non-human animals.

**Abstract:**

This review presents and analyzes recent scientific findings on the structure, physiology, and neurotransmission mechanisms of transient receptor potential (TRP) and their function in the thermoregulation of mammals. The aim is to better understand the functionality of these receptors and their role in maintaining the temperature of animals, or those susceptible to thermal stress. The majority of peripheral receptors are TRP cation channels formed from transmembrane proteins that function as transductors through changes in the membrane potential. TRP are classified into seven families and two groups. The data gathered for this review include controversial aspects because we do not fully know the mechanisms that operate the opening and closing of the TRP gates. Deductions, however, suggest the intervention of mechanisms related to G protein-coupled receptors, dephosphorylation, and ligands. Several questions emerge from the review as well. For example, the future uses of these data for controlling thermoregulatory disorders and the invitation to researchers to conduct more extensive studies to broaden our understanding of these mechanisms and achieve substantial advances in controlling fever, hyperthermia, and hypothermia.

## 1. Introduction

Thermoregulation plays a vital role in the survival of all endothermic organisms and altricial species, especially newborns [1,2,3,4]. Body temperature is a physiological and clinical parameter that provides relevant information on the individual’s state [5], while variations reflect valuable biomedical data [6]. Modulations of this parameter are closely related to the stability of numerous cardiovascular, respiratory, renal, endocrine, nervous, muscular, and cellular functions [7]. One example in humans is that the integrity of some cellular processes is altered within a specific range around the ideal temperature (37 °C) [8]. This characteristic of humans and non-human animals is known as the thermoneutrality zone (TNZ), which is defined as the environmental temperature range in which an organism does not need to activate metabolic and physiological pathways to dissipate or produce heat (heat loss, heat production) [9,10,11]. The precise TNZ range depends mainly on the species in question, physiological status (e.g., gestation), age, sex [12], body condition scores, and other factors that affect the thermoregulatory responses of the hypothalamus and preoptic nucleus [13,14].

Identifying between-species differences and determining the thermal comfort ranges of specific animal species is mediated by neurons in the central nervous system (CNS) and peripheral receptors [15,16]. Thermosensitive receptors exist in both prokaryotic and eukaryotic organisms [17]. The best-known central route of somatosensory cutaneous thermal signaling is the spinothalamic–cortical pathway, which originates in the activation of the system made up of thermoreceptors, thermosensors, and cutaneous effectors that carry signals of thermal stimuli to the dorsal root ganglion of the spinal cord. From there, the signals travel to the thalamus and, finally, to the primary somatosensory cortex, where body temperature is consciously perceived and integrated [18,19].

The sensory thermoreceptors in mammals are nerve endings with specialized ion channels that promote transitory modifications of membrane permeability, which depend on the external stimuli perceived [20]. Most of these are made up of transient receptor potentials that perform non-selective cation diffusion [21,22]; participate in the transduction of mechanical and chemical sensory stimuli and the maintenance of the membrane resting potential; and control calcium (Ca^2+^) and magnesium (Mg^2+^) levels in neurons and non-excitable or cancerous cells [23,24]. TRPs are expressed in almost all tissue cell types [25], excitable or non-excitable, and in all cell membranes except the nuclear and mitochondrial membranes. Most TRPs are located in the plasmatic membrane where they contribute significantly to numerous physiological processes and homeostatic functions, and participate in vasomotor control and muscular contraction [26]. Interestingly, although their structure has been studied in great detail, we still do not fully understand the mechanism by which TRP opens and closes its gates from the moment a thermal stimulus is perceived. Against this backdrop, the goals of this review are to analyze and contrast recent scientific findings on the morphology, physiology, and neurotransmission mechanisms of TRP, and their function in thermoregulation in non-human animals, as well as enhance our understanding of their functionality and fundamental role in maintaining the body temperature of animals and those susceptible to thermal stress—as can occur during transport [27,28,29].

## 2. Classification of the Ionic TRP Channels

The first step in the transmission of thermal stimuli, which later triggers the modulatory responses necessary to maintain thermoneutrality, involves the participation of the afferent nerve fibers whose free nerve endings hold TRP channels [30]. These channels, called *gene trp* initially, were first described in late 1960 in studies with fruit flies (*Drosophila melanogaster*) [17,26]. That research found that continuous luminous stimuli induced a transformation in the membrane permeability of photoreceptors that generated a severe state of blindness—although this was reversible after one minute spent in complete darkness [31]. This response was due to a signaling cascade that contained a transient current that allowed the entry of Ca^2+^, thereby producing a transitory change in receptor potential [32,33].

TRP are cation channels made up of transmembrane proteins [34] that function as transductors through changes in the membrane potential due to the intracellular concentrations of Ca^2+^ [35]. Q10 is the temperature coefficient of the rate of change when an organism increases its temperature by 10 °C. The normal value of Q10 is between 2–3, and in the case of biological processes or thermosensitive molecules, this value is usually more than 5. The thermosensitive TRP channels exhibit a specifically high Q10 coefficient that is higher or equal to 20 [17]. Generally, TRPs are cellular sensors that detect diverse environmental stimuli. Most respond to changes in temperature, pH, or osmolarity, but injuries, pain, pheromones, flavors, ionic imbalances (Ca^2+^), volatile chemicals, mechanosensation, and cytokines [36,37] can also activate them. According to the differences in their amino acid sequences and typological structures, TRPs are classified into groups, families, and subfamilies. Not all types respond to thermal stimuli, though all are involved in ionic regulation. From their discovery to date, some 30 *genes trp* and over 100 TRP channels have been recognized in mammals and have been divided into their corresponding families and subfamilies.

### 2.1. TRP Families and Subfamilies

**Figure 1:**
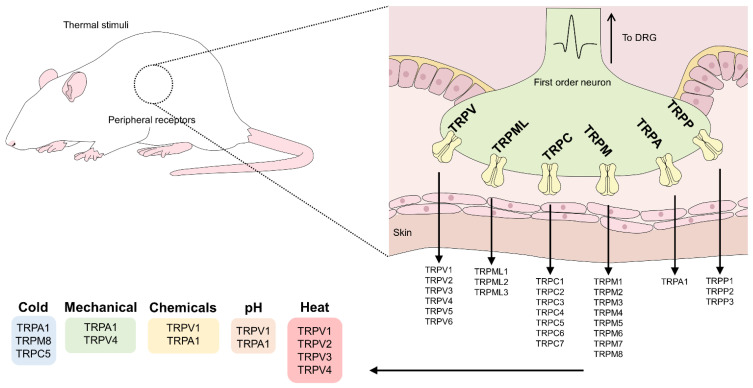
Families and subfamilies of TRP channels and their classification according to the type of stimulus they can perceive. DRG: dorsal root ganglion.

A total of 9 families [38] are recognized and divided into two groups depending on their degree of similarity with the *gene trp* found in *Drosophilas.* Group 1 (TRPN, TRPC, TRPV, TRPVL, TRPM, TRPS, and TRPA) includes the TRP with greater similarity to the gene, while group 2 contains those less similar (TRPP and TRPML) [23,30,32,37,39] (Figure 1).

#### 2.1.1. Classification of TRP

Activated in response to phospholipase C-coupled receptors and mechanical stimuli [40], TRP are divided into seven units [41]. Their characteristics include non-selective permeability to Ca^2+^ and a presence in several tissues, including the brain, gastrointestinal system, ovaries, endometrium, and ventricular myocytes. For this reason, their activation is also associated with emotional states like fear or stress [42].

#### 2.1.2. TRPV (Vanilloid)

This group consists of six units that are considered sensory mediators activated by endogenous ligands, heat, mechanical stimuli, and osmotic changes [43]. Of the six receptors included, TRPV1, TRPV2, TRPV3, and TRPV4 are called thermosensitive [44], while TRPV5 and TRPV6 are selective channels for Ca^2+^ ions whose function is to maintain Ca^2+^ homeostasis. TRPV are channels also involved in the transduction and activation of the nociceptive arch [45].

#### 2.1.3. TRPM (Melastatin-Related)

TRPM2, TRPM3, TRPM4, and TRPM5 are activated by heat. TRPM3 responds to harmful heat, while TRPM8 responds to cold temperatures [46]. One difference between TRPM4 and TRPM5 and the others is that they are not permeable to Ca^2+^, though they participate in membrane depolarization [47].

#### 2.1.4. TRPA (Ankyrin)

TRPA is the only element of this subfamily expressed in the primary somatosensory neurons, keratinocytes, astrocytes, smooth vascular muscles. and endothelium of the cardiovascular system, gastrointestinal and respiratory tracts, pancreas, inner ear, and odontoblasts [48]. It is considered a thermoreceptor sensitive to harmful cold temperatures and mechanical and sensory stimuli [49]. One of its main contributions is the transduction of nociceptive stimuli in the primary afferent nerve endings during signal transmission in the dorsal horn spinal neurons, but it also participates in episodes of neurogenic inflammation [48]. In clinical medicine, this receptor is being studied due to its activation and therapeutic effect under the administration of cannabidiol (CBD) [50].

#### 2.1.5. TRPN (No Mechanoreceptor Potential-C)

Mechanoreceptors are found only in zebrafish and invertebrates [51]. They are in charge of proprioception in species like *Caenorhabditis elegans* [52]. In the case of NOMPC *Drosophila*, only TRPNs are related to the thermoreception of harmful cold [38,53].

#### 2.1.6. TRPP (Polycystin)

These TRPs present in vertebrates and invertebrates respond to mechanical stimuli and the hydric balance and changes in intracellular Ca^2+^ concentrations given that non-selective channels are permeable to this compound [54].

#### 2.1.7. TRPML (Mucolipin)

TRPML, which is present in mammals and insects [38], are considered to participate in Ca^2+^ reuptake [52] and are sensitive to temperature changes.

#### 2.1.8. TRPVL (Vanilloid-like)

Described as a sister family to TRPV, these receptors are found only in animals belonging to the phylum Annelida (e.g., *Capitella teleta*) and Cnidaria (e.g., *Nematostella vectensis* and *Hydra magnipapillata)* [38,55]. It is suggested that this family arose as the last common ancestor of the phyla Bilateria and Cnidaria, but these receptors were lost in most bilaterians [56].

According to Peng et al. [55], TRPVL and TRPV channels share structural characteristics and, perhaps, similar functions.

#### 2.1.9. TRPC (Canonical)

These channels are known as critical ion channels for phototransduction [32] and were first described in flies [57]. They are classified from TRPC1 to TRPC7. TRPC1 was initially found in the brain, liver, and kidneys of fetuses, and the heart tissue, testes, ovaries, and brains of adults [58]. TRPC4 and TRPC5 are non-selective cation channels that can form homomers and heteromers, and are expressed mainly in the amygdala and hippocampus. Also, these channels are expressed in the peripheral sensory neurons of rodents [59] and humans [60]. TRPC5 is considered a cold-sensitive receptor [17], but, because TRPC5 also regulates prolactin release, TRPC5 antagonists may have greater analgesic efficacy in women since prolactin promotes pain only in them. Therefore, centrally-acting TRPC4 and TRPC5 antagonists could be an analgesic alternative to visceral and neuropathic pain [61]. On the other hand, although TRPC6 is highly expressed in the kidney, its role in kidney disease is complex and has hindered the development of some drugs aimed to improve kidney function [62].

#### 2.1.10. TRPS (Soromelastatin)

These receptors are considered one of the most recently discovered families and the least studied (functions and structure still unknown). These receptors are not found in insects or vertebrates; however, their presence has been reported in mollusks, tardigrades, nematodes, myriapods, and chelicerates [63].

#### 2.1.11. TRPY (Fungus-Specific TRP Channel)

Phylogenetic information has led to classifying TRPY as a different group from the previously described Group 1 and Group 2. Although the structure of this family is unknown, it is believed that these receptors may be a sister group to TRPP, which, like TRPY, has components of yeast [38,64]. TRPY has only been found in fungi and various physiological and pathological processes [65]. Likewise, it has been associated with oxidative and hyperosmotic stress, as well as in glucose-induced Ca^2+^ signaling. However, the exact function of the TRPY1-mediated Ca^2+^ release is still inconclusive [66].

### 2.2. Temperature-Sensitive TRP

Six subfamilies are identified as thermosensors (TRPV1-4, TRPM2-5,8, TRPA1, and TRPC5). Their firing frequency depends on the temperature they are exposed to (heat or cold) [19]. Neuronal afferents that perceive heat and cold can be differentiated by presenting specific TRP channels for each stimulus, and because their activation depends on specific thermal properties [67]. Most neurons that allow the identification of differences in the wide range of extreme temperatures (−10 to 60 °C), due to the thermosensitive receptors located in their nerve endings, are found in the trigeminal ganglion that innervates the face and head, and in the ganglia of the dorsal horn of the spinal cord [46]. According to the properties of the TRP thermosensors, these neurons recognize four basic thermal sensations: (1) cold, −10 to 15 °C; (2) cool, 16–30 °C; (3) warm, 31–42 °C; and (4) hot, 43–60 °C. Extreme cold and hot values are considered harmful stimuli [19]. The sensitive receptors to high (hot) temperatures are activated when they perceive a range of 23–52 °C [68]. They describe the thermal sensation as warm, hot, or harmful heat (pain) [30], while the ones that detect low temperatures (−10 to −42 °C) classify the thermal sensations as cool, cold, icy, or harmful cold (pain) [30,67].

**Figure 2:**
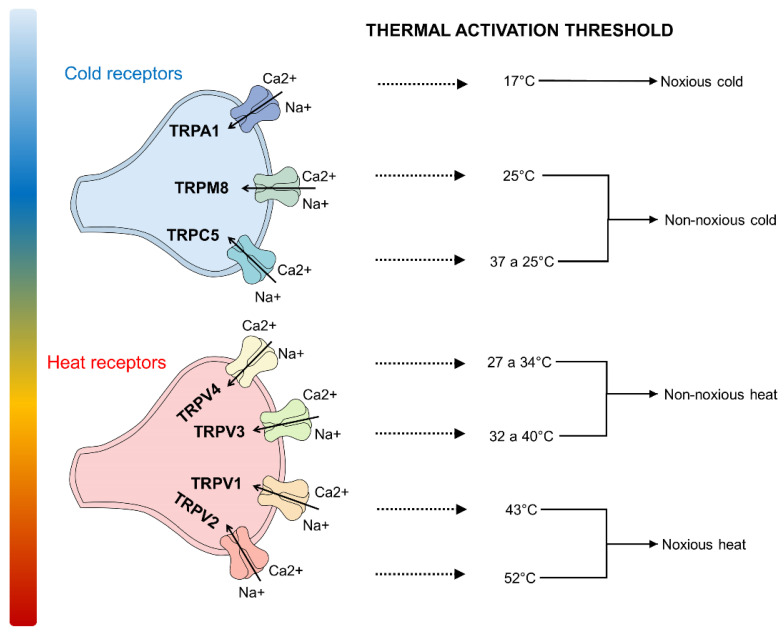
Thermosensitive TRP channels. Two types of thermoreceptors are known in mammals: those activated with warm or hot temperatures (range 27–52 °C) and can perceive harmful and non-harmful heat, and those whose activation threshold depends on cold stimuli (range 17–25 °C). Ca^2+^: calcium; Na^+^: sodium.

Four channels of the TRPV subfamily are recognized as heat-sensitive receptors. They, in turn, are divided into those activated by harmful heat (TRPV1 at 43 °C; TRPV2 above 52 °C) [68] and those activated by harmless heat (TRPV3, 23–39 °C; TRPV4, 27–39 °C) [69,70,71]. Those associated with harmful cold are TRPA1 [72,73], while the ones activated by non-harmful cold are TRPM8 and TRPC5 [23,30,32,46,74] (Figure 2). TRPM2, TRPM3, TRPM4, and TRPM5 also have thermosensitive properties but are not included among the thermoreceptors because they are not located in the primary afferent axons and, therefore, require specific concentrations of Ca^2+^ for their thermal sensitivity to be activated [17].

Although, functionally, all the TRPs exhibit differences that improve our understanding of the molecular bases of thermal stimulation [17], they all share a common morphology, as will be discussed later.

## 3. Structure

**Figure 3:**
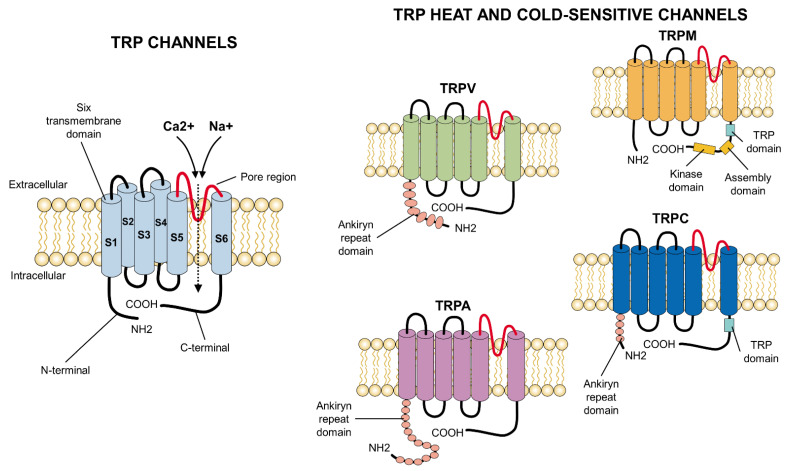
Primary structure of TRP ion channels. In general, all TRP channels are located in the cellular lipid bilayer and consist of six transmembrane subunits (S1–S6) with a pore region considered fundamental for the influx of cations, especially Ca^2+^ or Na^+^. The particularities which appear at the carboxyl- (C-terminal) or amino-terminus (N-terminal) (e.g., ankyrin repeats at the latter) differ among the families of TRP channels and confer specific properties to each receptor. COOH: carboxylic acid group; NH2: amino group.

TRP channels have a common primary structure similar to that of K^+^ channels. They consist of 4 subunits surrounding an ionic permeation [75]. Each subunit has six segments, or transmembrane domains [76], of which two (S5, S6) constitute the central pore, while the others (S1–S4) form a tetramer around it [74,76]. They contain long amino (N) and carboxyl (C) groups [33], which are located intracellularly. Since each subfamily of the TRP presents particularities in its soluble domains, a functional variability in each one can be distinguished [75] (Figure 3). TRP channels also have differential domains that have led them to be classified in distinct subfamilies whose morphological variations are based on comparisons of their amino acid sequences [77].

Most of the channels responsible for modulating temperature are located in the somatic and visceral afferents. The cold receptors are small diameter, myelinated axons (A-delta) in primates, but C fibers in non-primate mammals [67]. The cold-sensitive channels include TRPC5, whose structure is similar to that of the other members of the TRPC family and consists of six transmembrane helices adhered to the ends of the N- and C-terminal domains in spiral form, a TRP domain, ankyrin repeats (around 3–4), and hydrophobic pores in the form of a loop [35]. The TRPM8 channel is also important in cold thermosensation. Its structure is similar to TRPV1 with six helicoidal segments, of which S5 and S6, which are added to the helix of pores, form the pore domain. Its cytoplasmatic region comprises the C-terminal and D-terminal domains. The latter includes four melastatin zones [78]. The TRPA1 channel is another cold sensor. It contains a transmembrane domain (composed of six helixes with a loop between S5 and S6), an N-terminal domain that presents 14–17 ankyrin repeats, and another cytosolic C-terminal; it lacks TRP domains [35].

The axons that transmit heat stimuli function through unmyelinated C fibers, which are predominant in somatic and visceral tissues [67]. These channels include TRPV1, which respond to temperatures identified as harmful. TRPV1 has a quadruple structure composed of a compact zone that occupies approximately 30% of the total volume and an open domain in a somewhat basket-shaped form that makes up the other 70% [79]. TRPV2 has structural similarities to TRPV1 since both have two constriction regions—wherein one is in the S6 helix (distal zone) and the other in the selectivity filter. However, TRPV1’s pore is narrower than TRPV2’s, which is of complete length and, hence, capable of partially accommodating hydrated cations and other large organic ions [35].

Other important channels for heat sensitivity are TRPV3 and TRPV4, which are activated by non-harmful temperatures. The architecture of TRPV3 is similar to the TRPV channels, as it has transmembrane ionic channel domains constituted by S1–S4, amphipathic helixes, and pores added to an intracellular skirt domain that connects to the ankyrin repeats and encapsulates a cytoplasmic cavity. However, differences in its transmembrane ionic channel and ankyrin repeat domains can be observed. In addition, it presents a looped C-terminal domain that has not been seen in the other members of its subfamily [80]. TRPV4’s structure differs from TRPV1 in the pore, which has only one constriction in the narrowest region (lower gate) and lacks the upper gate. In addition, its selectivity filter is wider, and the turn that its S1–S4 domains make is in a clockwise direction and, concerning the S4 helix, at 90° [81].

## 4. Neurophysiology

The proteins that form the cell membrane channels regulate the influx of ions between the cell and its environment. Therefore, they have differential permeability. The main differences in the channels involve opening/closing mechanisms, conductance, dependence on membrane potential, kinetics, and other regulatory elements [82]. The mechanisms that these receptors utilize function simultaneously; that is, while the TRPV1 channels are activated by harmful temperatures above 42 °C, the TRPM8 close at temperatures over 33 °C and are activated only when temperature reductions below −15 and −30 °C are detected [46], which allow the entry of ions (Ca^2+^, Na^+^) that depolarize the membrane and initiate its action potential [17]. The morphology, physiological functioning, and cation permeability are similar in the nine subfamilies of the TRP channels that transmit signals to the CNS by opening gates [76]. The signaling mechanism can be direct or include the participation of second messengers [83].

The TRP channels are divided into three groups according to their permeability to Ca^2+^ or Na^+^: (1) TRP permeable to Ca^2+^ but non-selective, where the ion flow is mediated by Na^+^ and Ca^2+^ (TRPA1, TRPV1, TRPM3); (2) TRP selective to Ca^2+^, where the ion flow is linked only to Ca^2+^ (TRPV5, TRPV6); and (3) TRP impermeable to Ca^2+^ (TRPM4, TRPM5), where the ion flow is mediated primarily by Na^+^ [33]. Studies show that the central pore of the structure of the TRP is responsible for Ca^2+^ selectivity. The changes in the pore determine the differences in selectivity among the various types of TRP [84].

**Figure 4:**
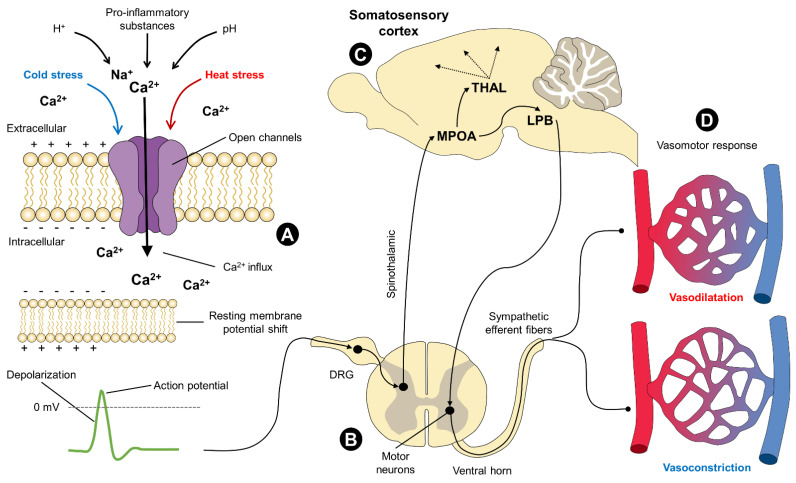
Activation and physiological participation of TRP channels in vasomotor thermoregulation. TRP receptors are channels permeable to Ca^2+^ and other cations (Na^+^). When they respond to cold or heat stress, changes in pH, the presence of H^+^, or pro-inflammatory substances, these channels open and allow the influx of Ca^2+^ into the intracellular space. The entry of cations into the membrane induces a shift in the resting membrane potential towards a more positive value. This change creates a depolarizing effect and the subsequent generation of action potentials. Once the perceived stimulus is transduced into an electrical signal, it is transmitted by primary neurons to the DRG of the spinal cord. For example, through ascending pathways (spinothalamic) signals are conveyed to brain centers essential for thermoregulation in mammals. The hypothalamus (MPOA), its connections to the thalamus, and the somatosensory cortex constitute the pathway of thermal perception. Vasomotor responses, meanwhile, are carried out through sympathetic efferent fibers that originate in the ventral horn of the spinal cord. Vasodilation and vasoconstriction are two physiological mechanisms that act to dissipate or conserve heat under exposure to hot and cold temperatures, respectively. DRG: dorsal root ganglion; LPB: lateral parabrachial nucleus; MPOA: median preoptic area; MV: millivolts; THAL: thalamus.

Since the TRP are non-selective cation channels and belong to the same family of voltage-gated Na^+^, K^+^, and Ca^2+^ channels, their activation triggers depolarization that, in turn, affects channels dependent on Ca^2+^ or those that contain it [85]. One of the principal characteristics of the TRP channels is that they function as signalers of intracellular Ca^2+^. By extracting it from the cytosol to generate an influx of cations and the onset of electrical activity, the generation of an action potential, and, finally, the physiological thermoregulatory responses that are activated depending on the stimulus perceived [33,86] (Figure 4).

### 4.1. The Opening/Closing Mechanism of TRP Channels

Recently, significant advances have been made to understand the structure of these channels. However, we do not yet completely understand the nature of the mechanisms through which their gates’ opening and closing operations function. In general, the activity of TRP channels requires post-transcriptional modifications such as the mechanisms related to the G protein-coupled receptors, dephosphorylation, and ubiquitination. The opening of channels selective to Ca^2+^ increases the amount of cytosolic Ca^2+^ and causes depolarization of the membrane, which results in an action potential and propagation towards the CNS [87]. This contributes to impulse transmission and generates the secretion of neuropeptides involved in neurogenic inflammation and other processes that depend on the activation of these receptors through diverse mechanisms [33].

#### 4.1.1. Membrane Voltage

A relevant number of TRP channels, most of them involved in sensory perception, have an intrinsic dependence on voltage [88,89]. Generally speaking, the influx of Ca^2+^, or under conditions in which large amounts of Ca^2+^ exist, generates an action potential in the membrane that makes it more positive. The opposite effect occurs when the membrane returns to a negative potential, thereby causing the inactivation of most TRP channels [90]. The voltage-dependent activation of the TRP is sensitive to other triggers, such as the presence of ligands or temperature changes [91,92]. Similar to what happens with the voltage-gated potassium channels, the molecular counterparts involved in detecting voltage are likely positively charged with lysine and arginine residues in the transmembrane segment, S4, and linker, S4 and S5 [92].

#### 4.1.2. Membrane Phospholipids

Studies mention that one route through which the TRP can be activated is mechanical stimulation, which causes a curvature in the layer and the opening of the channel due to ankyrin chains [76]. Several reports mention a direct effect of the membrane phospholipids on the regulation of TRP channel activity [93,94,95]. Specifically, many TRPs are highly sensitive to phosphatidylinositol 4,5-bisphosphate, which is the most abundant acid phospholipid in the plasmatic membrane [96,97].

#### 4.1.3. Phosphorylation

Protein kinases are a type of enzyme that modifies other molecules through phosphorylation. Phosphorylation that isdependent on the protein kinase C (PKC) is likely a direct mechanism of the channels’ activation, or sensitization, towards other stimuli. However, PKC reduces the function of some channels as its activation initiates dephosphorylation of TRPM8, thereby causing the inactivation of this channel [26]. Another mechanism of the activation of TRP channels involves the protein kinase A (PKA), whose activation through stimulation of the prostaglandin E2 potentializes TRPV1’s responses and counteracts the desensitization of that channel [98]. PKA and PKC present opposed mechanisms in modulating heat-activated TRPV1 and cold-activated TRPM8 [26,99].

#### 4.1.4. Ligands

Some activities of the TRP channels are regulated by a considerable number of exogenous and endogenous ligands [89], especially the ones that perceive thermal sensations, which seem to be the ones preferred by the chemicals derived from plants. Specifically, TRPV1, TRPV3, TRPM8, and TRPM4 depend on ligands to generate an action potential [90]. An example is TRPV1, which is activated by botanical compounds like capsaicin [100], resiniferatoxin, piperine, and camphor (the latter also activates TRPV3). TRPM8 is activated by menthol and eucalyptol [101]. In addition to the components of plants that can intervene in the activation of certain TRP, diverse synthetic ligands are utilized to activate these channels. They are essential pharmacological tools used to modulate TRP channels’ functions; for example, 2-aminoethyl diphenylborinate activates TRPV1, TRPV2, and TRPV3 [102], and icilin activates TRPM8 and TRPA1 [26,103].

## 5. Heat-Sensitive TRP

### 5.1. Harmful Heat

#### 5.1.1. TRPV1

This channel is responsible for perceiving harmful heat above 43 °C [19]. A non-selective channel that is permeable to cations, TRPV1 is activated by capsaicin, low pH (<6), temperature increases, and changes in membrane polarity [100]. It thus contrasts to TRPM8, which is activated by cold and menthol [17,104]. TRPV1’s activation range is 43–50 °C. Substances like ethanol also activate endogenous lipids (e.g., endocannabinoids, anandamides, and N-arachidonoyl dopamine), which are products of the lipoxygenase metabolism and topical analgesics. It is also associated with the recognition of harmful stimuli [17].

It is important to point out that diverse inflammatory mediators can reduce the temperature threshold for the activation of this thermoreceptor while increasing the magnitude of the responses of the ionic channel to produce inflammatory pain and heat hyperalgesia. For this reason, studies recommend that TRPV1 be a target for the development of analgesics [46]. In addition to its location in the sensory neurons of the dorsal root ganglia, TRPV1 is present, to a lower degree, in some cerebral structures, smooth muscle cells, adipocytes, the vascular system, and the gastrointestinal tract. Due to these circumstances, it has not yet been possible to precisely elucidate which sites TRPV1 contributes concretely to thermoregulation, for it also performs a function in regulating metabolism [46,105].

The observation that some TRPV1 knockout mice showed slight difficulties in detecting hot temperatures suggests that other mechanisms participate [106]. Reports describe the existence of a variant of the TRPV1 receptor in the sensory neurons of vampire bats that responds to a lower temperature threshold (~30 °C) and has been correlated with a delicate sensitivity that allows detection of warm-blooded prey [46,107].

#### 5.1.2. TRPV2

TRPV2 is considered a receptor present in A-delta heat-sensitive fibers, whose activation is observed at harmful temperatures above 52 °C [19]. It is also associated with the perception of osmotic stress to mechanical stimuli and pharmacological compounds, like cannabidiol and tetrahydrocannabinol [17]. It is found in pulmonary tissue, the spleen, intestines, dorsal root ganglia, sensory ganglia, and the brain. TRPV2 is the principal receptor expressed in cerebral tissues (e.g., cerebellum, forebrain, and hippocampus). It has been suggested that in addition to its thermo-transductor functions, it may participate in the physiological processes in those cerebral structures [108]. Although TRPV2 presents 50% homology with TRPV1, it is not activated by capsaicin and is more permeable to Ca^2+^ than Na^+^ [36]. The role of this thermosensor has been studied in heat-sensitive nociceptors of rats, where administration of drugs like gadolinium—a TRPV2 antagonist—blocked the influx of cations, thus restraining the activation [108]. In contrast, the 2-aminoethoxydiphenyl borate agonist generates action potentials in the receptors in rats and mice, but not in humans [109]. Despite these findings, doubts persist concerning the participation of this receptor in thermoregulation because studies of TRPV2-deficient mice have not reported any aberrant thermosensitivity [110].

### 5.2. Harmless Heat

#### 5.2.1. TRPV3

TRPV3 is a cation channel activated at warm temperatures around 34 °C [19], and ranging from 32–40 °C [111]. It belongs to the permeable channels group that are non-selective for Ca^2+^. It is expressed in the skin, keratinocytes, and oral and nasal mucosa, where its activation participates in other processes unrelated to thermosensitivity—such as hair growth, wound healing, itching, and pain perception [112]. Its presence in dermal tissues and the process of hair growth in mammals has led to studies of its participation in the processes of alopecia in hairless rodents. In those animals, mutations generate the absence of hairy structures due to an ionic alteration in the skin and observations of non-responsiveness to thermal stimuli and harmful chemicals [113]. Similarly, experimental results from Ferreira et al. [17] and Moqrich et al. [101] report that mice with genetic deficiencies of this receptor show altered responses to both harmless and harmful thermal stimuli. In contrast, Huang et al. [114] and Miyamoto et al. [115] mention that the TRPV3 and TRPV4 channels in mice do not play a fundamental role in heat perception or processes of heat hyperalgesia since animals deficient in this ionic channel did not manifest a deficit in their responses to heat stimuli. Variability in these results suggest that these phenotypes depend significantly on the genetic background of the mice tested [46].

#### 5.2.2. TRPV4

This is a non-selective polymodal receptor similar to TRPV1 that is activated by moderately warm temperatures in the range of 27–34 °C [19]. In mammals, exposure to this temperature range, and the subsequent activation of TRPV4, generate the influx of Ca^2+^ and an action potential that helps them maintain thermal homeostasis concerning their environment or external stimuli [116]. Other functions of TRPV4 consist of acting as a sensor of hypotonicity [111] and as a mediator of such pathological processes as heat hyperalgesia and mechanical hyperalgesia. In both cases, excessive excitation due to direct damage, like spinal cord compression, leads to nitric oxide generation and a cascade that triggers sensitization processes like hyperalgesia and allodynia [117].

Other examples have been observed in species like mice when the TRPV4 gene is experimentally removed. This procedure revealed a deficit in perception of mechanical and chemical stimuli (tail pressure and the acetic acid test, respectively). However, those same subjects’ thermal responses to harmful heat (50 °C) were not altered, suggesting that one of TRPV4’s main roles is to perceive nociceptive mechanical stimuli rather than participating in thermal sensibility [118]. Like TRPV3, the fact that TRPV4 is heat-activated suggests that it participates in maintaining body temperature; however, no clear evidence exists to support this hypothesis [46].

#### 5.2.3. TRPM2, TRPM4, and TRPM5

Since these three receptors require modulation by intermediaries (e.g., ADP-ribose, intracellular Ca^2+^, oxidative stress), and because they are mainly located in the brain, pancreas (TRPM2 specifically in β cells), and immune system cells, they tend to be excluded from the list of primary thermosensitive receptors [17,119]. Nonetheless, some studies have demonstrated their importance as thermal sensors involved in events like fever, where they intervene to prevent hyperthermia and its organic consequences, and their participation in hypothermia development since TRPM2 activation and inhibition modulate body temperature [120]. However, no thermosensory phenotype has been described in mice whose TRPM2 or TRPM4 receptor is inactivated. TRPM5 has been associated with the modulation of taste perception [121], but it remains unknown whether this characteristic acquires an important role in other homeostatic contexts [46].

## 6. Cold-Sensitive TRP

### 6.1. Harmful Cold

#### TRPA1

This receptor responds to a thermal threshold of 17 °C, which is considered harmful cold [19]. Increased intracellular Ca^2+^ concentrations mediate its activation due to the cold more than any direct action. Significantly, depolarization of TRPA1 is considered a fundamental element for inducing the cutaneous vasoconstriction responses that occur when individuals are exposed to low temperatures [122]. Some studies have found differences between species in which cold temperatures have activated TRPA1, like in rats and mice but not in humans or Rhesus monkeys [123]. Moreover, contrasting results have been reported within the same species (mice) since some authors indicate that this receptor contributes to the sensation of cold [124,125], but others rule out any such participation in detecting cold in vivo [126,127] or in mediating cold defense responses [128,129].

This receptor’s responses to harmful thermal (heat) and chemical stimuli have been studied in frogs and lizards, like the green anole (*Anolis carolinensis*). Saito et al. [130], for example, found that TRPA1 is activated under exposure to both conditions. A finding related to an evolutionary effect in which those species have preserved the TRPA1 channel for the detection of temperatures that significantly alter their homeostasis and, in this way, increase their probability of survival. Similarly, TRPA1 is associated with the transduction of nociceptive stimuli, like those linked to pro-inflammatory transmitters, such as the bradykinins [131].

### 6.2. Harmless Cold

#### 6.2.1. TRPM8

This ion channel was the first one to be described as a thermoreceptor that specialized in the thermal sensation of cold. Activation of TRPM8 occurs under exposure to temperatures below 25 °C [19]. However, some authors mention that its activation begins at around 33 °C in the sensory neurons and that its signaling velocity increases proportionally to the temperature decrease [17,104,120]. This receptor is present in a subset of afferent neurons in the dorsal root ganglia that innervate the skin and sensory neurons of the trigeminal ganglion that innervate the head, eyes, and cornea [46]. There are also reports that TRPM8 is expressed in adipocytes, as mentioned with regards to TRPV1 [132,133].

Scientific evidence indicates that diverse, selective TRPM8 antagonists produce dose-dependent hypothermia in rats and mice. The topical application of menthol, a TRPM8 agonist, triggers hyperthermia and shivering-like muscle activity, vasoconstriction at the level of the skin of the tail, and heat-seeking behavior [134]. These findings concluded that there is a hypothermic effect dependent on this receptor’s activity [46].

#### 6.2.2. TRPC5

TRPC5 is considered a cold-sensitive receptor that is activated at temperatures of 37–25 °C. It is present in the neurons of the dorsal ganglia and the dorsal lamina of the spinal cord [17]. Despite reports that describe the cold-induced gating of this receptor, studies of mice after ablation of the TRPC5 gene have concluded that those subjects do not present detectable defects related to cold sensitivity. Those findings call the role of this ion channel into question [46].

## 7. Uses of TRP in Disease Treatment

Due to the variety of functions in which the TRP channels participate, and given the diversity of mechanisms that exist for their activation, failures could occur in the permeation or entrance of a channel that would have a considerable influence on the progress of various illnesses. The above reflects that associations have been found with affectations of the intestinal, renal, urogenital, respiratory, and cardiovascular systems, and in neurodegenerative and neuronal diseases [135]. It is also important to note that alterations of the TRP channels can affect the progression of tumors in both early and late stages, and that they have been related to diverse types of cancer, including melanoma, glioma, prostate, bladder, mammary gland, and kidney cancer [136].

The ones susceptible to thermal changes stand out because they significantly influence homeostatic processes [135]. One example of the influence of these temperature-sensitive channels can be appreciated in the study by Hoshi et al. [137], in which the authors induced edema in vitro by an ischemic stroke. Their analysis of thin slices of brain tissue under conditions of oxygen–glucose deprivation revealed that this form of deprivation-induced inflammation had been activated by the temperature increase that caused hyperthermia and generated inflammation as a consequence—but they also found that the inflammation could be blocked by administering drugs that genetically annulled TRPV4.

A study by Mustafa and Ismael [138] utilizing 20 New Zealand white rabbits isolated the carotid arteries and suspended them in organ baths, to which they added portions of ethanol, capsaicin, and capsazepine. They aimed to explore the relationship between ethanol and hyperthermia accompanied by vasoconstriction of the carotid valve. They succeeded in determining that the increase in temperature provoked by ethanol-generated vasoconstriction of the carotid valve was due to this process, which is mediated by activation of a present TRPV1 by the temperature increase and capsaicin. These findings are significant because identifying the participation of this channel and implementing antagonists that inhibit it (like capsazepine) can help prevent heat stroke or brain damage caused by hyperthermia and the decrease in cerebral perfusion induced by ethanol. A study by Tan and McNaughton [139] concluded that in addition to playing an important role in thermoregulation, TRPM2 intervenes in controlling the immune system, pain, and insulin secretion; however, its action mechanism is not clearly understood. Meanwhile, the results of Di et al.’s study [140] suggest that TRPM2 plays a negative role in the feedback of the buffering of inflammatory responses induced by oxidative stress. Beceiro et al. [141] demonstrated that in mice chronically infected with *Helicobacter pylori,* TRPM2 increased gastric inflammation and the production of macrophages and inflammatory mediators, which was accompanied by the polarization of M1 macrophages. The evidence from these studies concludes that TRPM2 has an essential role in immune system processes that are known to be closely linked to temperature, though controversy continues as to whether it intervenes favorably or unfavorably in inflammatory processes.

Other studies have shown that the activation of cold receptors in mice in environmental temperature conditions below the TNZ of the species provokes metabolic expenditures and energy demand to achieve thermogenesis. These changes entail a risk for animals, especially laboratory species, in which a disfunction of receptors increases the incidence of cardiovascular and autoimmune diseases, asthma, and cognitive diseases like Alzheimer’s [14].

This discussion emphasizes the importance of determining and understanding the functioning of TRP and thermoregulation in the field of medicine [142], for this could contribute to developing new therapeutic strategies to treat specific pathologies. One example comes from work on TRPV3, a receptor mainly expressed in the skin and involved in skin health. At the experimental level, antagonists of this thermoreceptor have been used to treat dermal inflammatory pathologies because activation of TRPV3 generates secretion of proinflammatory, pro-nociceptive, and pruritic substances [143]. Due to the direct participation of TRPV3 in the process of nociceptive arch transduction, it has been proposed as an option for treating and preventing pain, though its functionality as an analgesic agent is not firmly established [144]. Finally, regulation of this receptor has been shown to affect nitric oxide production in keratinocytes that, in addition to participating in inflammation and synaptic plasticity, modulate vascular tone and, with this, blood flow and the amount of heat that a body dissipates [115].

Although currently there are analgesic options such as non-steroidal anti-inflammatory drugs [145] or opioids [146], novel therapies aimed to target specific TRP channels can contribute to modulating pain through processes such as desensitization [48]. Since most TRP channels are located in the periphery to detect noxious stimuli, drugs that target TRPs serve to block the transduction of the nociceptive stimuli [147]. For example, TRPV1 located in nociceptors is characterized by a rapid activation by noxious thermal stimuli, followed by a rapid –heat-induced desensitization [148]. This property allows the use of therapies with capsaicin patches, capsaicin analogs, and TRPV1 agonists such as resiniferatoxin (RTX). Due to its long-lasting refractory period that results in desensitization and prolonged analgesic activity [149], target therapy to TRPV1 with RTX has been suggested as a clinical alternative for severe osteoarthritic [150] or cancer pain, where its mechanism of ablation of TRPV1 channels is through calcium-induced apoptosis without generating somatosensory consequences [151].

## 8. Areas of Opportunity

Although the discovery of TRP channels has revolutionized our understanding of diverse physiological and sensory processes, this review highlights numerous unresolved issues. An example is the levels of participation of receptors like TRPV1, TRPV2, TRPV3, and TRPV4, since some studies report that, at least in certain mouse species, their antagonism or absence does not seem to trigger any aberrant thermosensitivity. For this reason, it is necessary to continue researching to understand these mechanisms in their totality. That knowledge could be used to control thermoregulatory disorders and develop drugs that can help improve the management of alterations caused by failures in the functioning or activation of these channels through the use of antagonists that inhibit them, given that several of them potentiate inflammatory processes. Moreover, an improved understanding of the functioning of TRP channels can contribute to adapting measures capable of mediating processes like fever, hyper-, and hypothermia.

Furthermore, TRP channels are not the only ones involved in the perception of thermal stimuli. Studies of lipid-sensitive TWIK-related potassium channels constitute another field of interest related to thermoreceptors. These channels respond to hot and cold temperatures in an activation threshold of 25–37 °C. This way, they have been associated with normal physiological maintenance and functioning through the conductance of K^+^. They can be activated by mechanical stimuli, pH, unsaturated fatty acids, and general anesthetics. Upon receiving harmful thermal stimuli, they generate hyperpolarization that reduces their capacity to send signals, leading to a type of heat-pain relief [19]. Meanwhile, the non-selective cationic channels of the HCN family (HCN1) participate in firing patterns, especially during cold sensations. Mice with a deficiency in the presenting genes of these receptors show altered responses to perceptions of cold [17].

The presence of these non-TRP thermosensitive channels also influences the response of knockout animals whose TRP function is reduced. In this sense, Wang and Siemens [46] mention that the lack of a single TRP channel does not always translate into an immediate deficiency due to the synergism of other TRP and non-TRP channels that can be activated by overlapping thermal sensations (e.g., the interaction between TRPV1, TRPM3, and anoctamin 1). For example, TRPM3 is a polymodal heat thermosensor with similar functionality as TRPV1 that acts independently of the vanilloid family [152]. Likewise, Vandeuwauw et al. [153] reported that only TRPV1, TRPM3, and TRPA1 triple knockout mice completely lacked the response to noxious heat stimuli, showing that the processing of a thermal stimulus is not dependent on a single receptor but instead requires the co-expression of other thermosensitive channels. The chloride channel Anoctamin 1 (ANO1) is another receptor activated by noxious heat [154] that responds to temperatures over 44 °C [155]. In ANO1, an interaction with TRPV1 and a similar inhibitory response have been reported when substances such as 4-isopropylcyclohexanol are administered [156].

Another area in which studies of TRP channels have focused is in developing and implementing rehabilitation therapies based on cold or hot temperatures. Cold therapies generate reductions in the degree of pain perceived in cases of skeletal muscle injury and reduced blood flow, thus decreasing local inflammation and the incidence of edemas [157]. In contrast, therapies that use ultrasound equipment with heat help reduce pain, aid in the recovery of muscular strength, and increase patients’ flexibility [158]. Therefore, these approaches could constitute options for managing skeletal muscle injuries in sports medicine.

As the reader can appreciate, research has focused on analyzing the activity of these thermoreceptors in species like rats, mice, and even bats. However, the information available on birds or ectoderms is scarce, so we invite researchers to design more studies with these animals to improve our understanding of thermoregulation in more species.

## 9. Conclusions

The participation of some TRP channels—like TRPV1-TRPV4, TRPM8, and TRPA1—in the reception of thermal sensations is still a subject where controversy exists regarding the mechanism through which thermal stimuli are detected, and the role they perform in the thermosensitivity of diverse species. This is due to two facts: first, that research has centered primarily on mammals, and second, that even within the same species there are reports of contrasting results. While TRPV1 and TRPV2 are sensitive to harmful heat, TRPV3 and TRPV4 perceive temperatures in the range of harmless heat, and TRPM8 channels are involved in the perception of harmless cold, but TRPA1 perceives harmful cold. Another aspect of the challenges we face is that although the structures and subtypes of TRP channels are now well known, their mechanisms of activation and regulation continue to present enigmas in both the field of habitual thermoreceptor mechanisms and research on the administration of certain drugs. Consequently, we currently lack the compounds, tools, and peptide toxins that would make it possible to capture TRP channels in distinct functional states. For these reasons, pharmaceutical companies’ research discoveries and developing medications must identify new molecular compounds, peptide toxins, or sensitive antibodies that can modulate the function of TRP channels.

In conclusion, it does not suffice for the pharmaceutical industry to conduct additional research in this field. It is also necessary to deepen our understanding of the action mechanisms of TRP channels to make better use of their applications in clinical medicine. Also, to prevent pain involving small animals, wild species, and farm animals, detect it opportunely, identify thermal stress situations, and even the onset of diseases that could participate in inflammatory or immune processes.

## Data Availability

No data collected.
